# Resolving Astrocyte Heterogeneity in the CNS

**DOI:** 10.3389/fncel.2017.00300

**Published:** 2017-09-27

**Authors:** W. Todd Farmer, Keith Murai

**Affiliations:** Centre for Research in Neuroscience, Department of Neurology and Neurosurgery, Brain Repair and Integrative Neuroscience Program, The Research Institute of the McGill University Health Center, Montreal General Hospital, Montreal, QC, Canada

**Keywords:** astrocyte-neuron interactions, astrocyte heterogeneity, Sonic hedgehog (Shh), notch, Fgf, Bergmann glia, Müller glia

## Abstract

Astrocytes play essential roles in nearly all aspects of brain function from modulating synapses and neurovasculature to preserving appropriate extracellular solute concentrations. To meet the complex needs of the central nervous system (CNS), astrocytes possess highly specialized properties that are optimized for their surrounding neural circuitry. Precisely how these diverse astrocytes types are generated *in vivo*, however, remains poorly understood. Key to this process is a critical balance of intrinsic developmental patterning and context-dependent environmental signaling events that configures astrocyte phenotype. Indeed, emerging lines of evidence indicate that persistent cues from neighboring cells in the mature CNS cooperate with early patterning events to promote astrocyte diversity. Consistent with this, manipulating Sonic hedgehog (Shh), Notch and fibroblast growth factor (FGF) signaling in the adult brain, have profound effects on the structural, morphological and physiological state of mature astrocytes. These pathways may become disrupted in various neurological diseases and contribute to CNS pathology. This mini-review article focuses on how context-dependent environmental cues cooperate with intrinsic developmental patterning events to control astrocyte diversity *in vivo* in order to promote healthy brain function.

## Astrocyte Diversity Across Brain Regions

Astrocytes are critical non-neuronal cells of the central nervous system (CNS) that maintain precise environmental conditions for neurons and their connections. The complex structural and molecular properties of astrocytes enable these cells to play major roles including extracellular ion maintenance, neurotransmitter recovery and regulation of cerebrovasculature. Of particular interest for this mini-review article is the protoplasmic astrocyte which has a sponge-like appearance and closely interfaces with neurons, synapses and cerebrovasculature. These cells are extremely complex, enveloping neuronal cell bodies and synapses with fine processes, and surrounding blood vessels with endfeet. These processes are loaded with specific molecular machinery allowing these cells to fulfill many functions including removing neurotransmitters and ions, secreting neuroactive substances that regulate synapses, regulating energy substrates and exchanging nutrients with vasculature.

**Figure 1:**
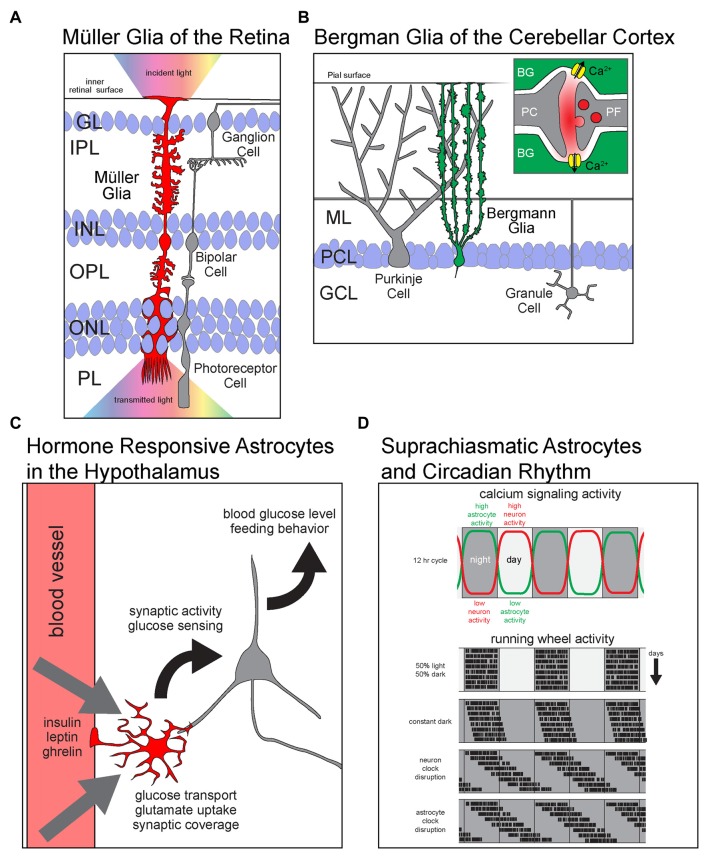
Astrocyte diversity across brain regions. Schematics showing region specific properties and functions of different astrocyte types. **(A)** Müller glia (MG) of the retina are radially polarized astrocytes that span all retinal layers. With endfeet attached to the inner retinal surface and soma in the inner nuclear layer (INL), they elaborate processes into every layer of the retina. MG facilitate the transmission of light (rainbow) from the inside of the eye (up) across the retina to the photoreceptors in PL. GL, granule layer; IPL, inner plexiform layer; INL, inner nuclear layer; OPL, outer plexiform layer; ONL, outer nuclear layer; PL, photoreceptor layer. **(B)** BG are radially polarized astrocytes that manage the glutamatergic synapse-rich neuropil of the cerebellar cortex. (**B**, inset) Glutamate released at PF-PC synapses activates calcium permeable ionotropic AMPA receptors and causes and influx of Ca^2+^ into astrocytes. PC, Purkinje cell; BG, Bergmann glia; ML, molecular layer; PCL, Purkinje cell layer; GCL, granule cell layer; PF, parallel fiber. **(C)** Astrocytes in arcuate nucleus of the hypothalamus are critical regulators of satiety and energy homeostasis. They respond directly to the hormones insulin, leptin and ghrelin by modulating glucose transport and the synaptic activity of hypothalamic neurons. Ultimately, astrocytic metabolic hormone signaling is necessary for the homeostatic maintenance of blood glucose levels and the regulation of feeding behavior. **(D)** Astrocytes of the dorsal suprachiasmatic nucleus (dSCN) are critical players in the maintenance of circadian rhythm. (**D**, upper panel) The levels of calcium signaling neurons and astrocytes are anti-phase; neurons are active during the day while astrocytes are active during the night. (**D**, lower panels) Simulated plots of periodic wheel running activity of mice under multiple conditions. The motor activity of mice is largely constrained to dark periods during equal length light and dark cycles. When kept in constant darkness, circadian period is only slightly shifted as revealed by their motor activity. However, when the intrinsic circadian clock of dSCN neurons is lengthened, the mice display a large shift in the periods of motor activity. Interestingly, when the same manipulation to lengthen the intrinsic circadian period is performed on dSCN astrocytes, the mice show a shift in behavior that resembles the neuronal manipulation.

Astrocytes were initially defined by their star-shaped morphology in the 19th century. Today, however, it is more greatly appreciated that astrocytes display an array of morphological and functional features that reflect the brain circuitry in which they surround. In some regions of the CNS, astrocyte morphology is so dramatically different that it can be used to identify the boundaries of anatomical regions (Emsley and Macklis, 2006). However, recent studies have shown that differences between astrocytes go well beyond their morphology, with some CNS astrocytes displaying distinct molecular and functional properties that are exquisitely matched to the function of neighboring neurons. Below are several examples of astrocytes from different CNS regions that possess distinct structural and/or functional properties with a clear adaption to nearby circuitry (Figure 1).

### Müller Glia and Retinal Function

Müller glia (MG) of the retina show a number of specific properties tailored to retinal function such as fluid transport and protecting the system from oxidative stress during photoreception (for review, see Reichenbach and Bringmann, 2013). MG are radially polarized with endfeet attached to the inner surface of the retina forming a parallel array of cells that span all retinal layers. Remarkably, MG processes projecting into each layer conform to the anatomical features of each layer of the retina. Hence, MG display one of the clearest examples of morphological adaptation of astrocyte shape and function to the local cellular and subcellular environment (for review, see Derouiche et al., 2012). Another distinctive function of the MG is to act as an optic channel to prevent light scattering as it passes through several neuropil and nuclear layers (Franze et al., 2007).

### Bergmann Glia in the Cerebellar Cortex

Like MGs, Bergmann glia (BG) of the cerebellar cortex are another example of an astrocyte that deviates from the classical star-shaped morphology. BG are morphologically polarized astrocytes with their soma closely associated with those of neighboring Purkinje cells (PC). Elaborate processes extend through the molecular layer (ML) and envelope synapses within the dense neuropil of the ML. Along with their unique morphology comes a specialized gene expression profile optimized for dealing with the dense glutamatergic innervation of the ML. BG express high levels of proteins used to detect and manage extracellular glutamate including the glutamate transporters EAAT2/GLT1 and EAAT1/GLAST as well as calcium permeable ionotropic glutamate receptors composed of GluA1 and GluA4 subunits. The expression of AMPA receptors is required for BG detection of glutamate and fine motor coordination (Saab et al., 2012).

### pH Sensitive Astrocytes in the Ventral Brain Stem

The brain stem serves many critical functions in the CNS including the regulation of homeostatic processes involved in breathing. Remarkably, astrocytes are important participants in respiration. Astrocytes of the chemoreceptive regions of the ventral surface of the medulla oblongata respond to changes in blood pH and release ATP in response high CO_2_ levels to adjust respiration rate. Ventral brainstem astrocytes have been shown to have a unique ability to sense/respond to pH, a property not found with dorsal brainstem astrocytes (Gourine et al., 2010).

### Hormone Responsive Astrocytes in the Hypothalamus

The hypothalamus is essential for regulating thirst and hunger. Astrocytes in the arcuate nucleus of the hypothalamus respond to the hormones leptin, ghrelin and insulin to influence neuronal activity, regulate glucose uptake across the blood brain barrier, and modulate the rate of food consumption (Kim et al., 2014; Fuente-Martín et al., 2016; García-Cáceres et al., 2016). Astrocytes in adjacent hypothalamic nuclei do not respond to these hormones, further indicating region-specific role of astrocytes in the arcuate nucleus.

### Suprachiasmatic Astrocytes and Circadian Rhythm

The dorsal suprachiasmatic nucleus (dSCN) is the central regulator of circadian rhythm. Cell-autonomous cycles of gene expression define the 24-h cycle in the absence of external cues. Recent findings show that astrocytes of the dSCN are critical regulators of the length of the circadian cycle (for review of recent findings, see Ruben and Hogenesch, 2017). dSCN neurons are active during the day whereas astrocytes of the dSCN are active during the night and serve to silence neuronal activity. The anti-phase oscillations of neuronal and astrocytic activity provide a mechanism to enforce a strict 24-h cycle. Lengthening the intrinsic circadian cycle of dSCN astrocytes results in an increased duration of the circadian period which is reflected in shifted sleep/wake behavior (Brancaccio et al., 2017).

The examples described illustrate extreme structural and functional properties that can be found for astrocytes in different CNS regions. However, major differences in astrocyte properties are likely wide-spread across brain regions and independent of morphological dissimilarities. For example, hippocampal and striatal astrocytes are significantly different in Kir4.1 currents, calcium signaling, encompassing volume and number of neurons/synapses within the territory of an individual astrocyte (Chai et al., 2017). Furthermore, astrocytes from different brain regions secrete variable amounts of synaptogenic factors and contain heterogeneous levels of glycogen (Oe et al., 2016; Buosi et al., 2017). Further investigations are needed to further identify differences among astrocytes in order to fully understand their overlapping and unique roles in the CNS.

## Developmental Patterning and Interregional Astrocyte Heterogeneity

Many of the differences between astrocytes likely spawn from distinct developmental programs which result in specific cellular identities for a given brain region. Given the need of astrocytes to fulfill specific support roles of diverse neuron populations it is not surprising that astrocyte heterogeneity mirrors neuronal diversity. But how are different astrocytes actually created throughout the brain? Astrocytes generally come from related progenitors as the neurons that they support. Thus, patterning and specification of astrocytes may follow the same fundamental principles as those guiding neuronal development within a given brain region. This might ensure that astrocytes acquire specific properties that are configured for the surrounding neuronal population.

### Intrinsic Programs Underlying Astrocyte Heterogeneity: Lessons from the Spinal Cord

The patterning events that give rise to the discrete domains and neuronal populations of the spinal cord are well characterized. Opposing dorsal-ventral gradients of morphogens such as Sonic hedgehog (Shh) and bone morphogenetic protein (BMP) segregate the spinal cord into defined progenitor domains. Each progenitor domain gives rise to sequential populations of neurons and astrocytes with specific properties and locations (Hochstim et al., 2008). The specification of neurons and astrocytes utilizing the same transcription factor code likely helps guarantee that astrocytes have fundamental properties that match the physiological needs of related neurons including the expression of cues that guide neural circuit formation (Hochstim et al., 2008; Molofsky et al., 2014). The region-specific allocation of spinal cord astrocytes appears to be an intrinsic property, as astrocytes are unable to populate adjacent domains where other astrocytes have been ablated (Tsai et al., 2012).

### Allocation of Cortical Astrocytes

Recent studies have begun to unravel how astrocytes populate the cerebral cortex, a brain region where heterogeneity of astrocytes is especially complex. Like the excitatory neurons of the cortex, cortical astrocytes are derived from progenitors that line the ventricular surface of the developing forebrain. However, astrocytes are produced from the neurogenic ventricular progenitors only after they have exhausted their neurogenic potential (Gao et al., 2014). In the cerebral cortex, newly born astrocytes migrate outward towards the pial surface and fill each of the cortical layers. Intriguingly, cortical astrocytes show little or no lateral migration outside of a domain that consists of a single cortical column (Magavi et al., 2012). Some clonally derived astrocytes, like neurons, are dispersed across cortical layers in a columnar pattern (García-Marqués and López-Mascaraque, 2012; Gao et al., 2014). However, the generation of astrocytes in the cortex differs from neurons in three major ways: (1) the relative time of birth of an astrocyte does not appear to determine its radial position, and (2) some astrocytes will divide symmetrically to locally produce more astrocytes, a process that continues into adulthood (Ge and Jia, 2016); and (3) a subpopulation of cortical astrocytes appear to be derived from NG^2+^ cells (Zhu et al., 2008).

## Environmental Cues and Intraregional Heterogeneity of Astrocytes

The patterning of the nervous system provides a mechanism to generate extensive cellular diversity. Interestingly, astrocytes can show diversity even within a defined CNS region. One of the most striking examples of intraregional heterogeneity is the stark difference between the two major astrocytes of the cerebellum, BG and velate astrocytes (VAs). BG and VAs are derived from the same progenitor pool (Kita et al., 2013), yet have contrasting morphologies and gene expression profiles. Remarkably, it is persistent signaling from adjacent neurons that is responsible for maintaining the unique gene expression profile of mature BG (Farmer et al., 2016). In other brain regions, astrocyte heterogeneity appears to be more complex. In the striatum, intermixed astrocytes and neurons from multiple progenitor pools interact indiscriminately yet astrocytes show selective interactions with specific circuits (Martín et al., 2015; Torigoe et al., 2015). Single-cell RNAseq of the striatum indicates that unlike other cells of the striatum, astrocytes do not segregate into discrete populations and therefore do not appear to form well-defined subtypes (Gokce et al., 2016). In the cerebral cortex, astrocyte can display diverse expression patterns coupled with layer-specific molecular heterogeneity. The intraregional heterogeneity of cortical astrocytes is easily revealed by immunolabeling for the inward-rectifying potassium channel Kir4.1, the GABA transporter GAT3/Slc6a11, the glutamate transporters EAAT1/GLAST and EAAT2/GLT1, and the water channel aquaporin 4 (AQP4). GAT3 and Kir4.1 are particularly enriched in Layers 2/3 and Layer 5 (Conti et al., 2004; Moroni et al., 2015), whereas Aqp4, glial fibrillary acidic protein (GFAP) and Connexin-43/Gja1 show strong expression in Layer 1 and in pial astrocytes in the mouse (Zeisel et al., 2015).

## Context-Dependent Signaling and The Expansion Astrocyte Diversity

How do intraregional differences in astrocytes arise? A creative solution is to enable astrocytes to use environment cues to configure their properties within a given brain region. This type of mechanism would ensure that astrocytes are precisely matched to neighboring neurons and associated circuitry and, moreover, be able to dynamically shift their functional and/or structural properties to adapt to new environmental situations. Recent studies have now found that components of multiple signaling pathways that pattern the developing CNS are enriched in mature astrocytes (Cahoy et al., 2008) and, importantly, persistent signaling through specific signaling pathways is required to maintain astrocyte properties in the adult brain. Culturing astrocytes with neurons causes increased astrocytic elaboration and the induction of hundreds of astrocyte genes including Glast, GLT1, AQP4 and GAT3 (Hasel et al., 2017).

### Sonic Hedgehog Regulates Astrocyte Diversity in Cerebellum, Cortex and Hippocampus

Recent work from our laboratory has shown that adult astrocytes depend on the neuron-derived factor Shh for configuring their molecular profile. Shh is a morphogen known for its role in ventralizing the developing CNS. In the adult brain, Shh is expressed by neurons while astrocytes express receptors and signaling components needed to respond to Shh (Cahoy et al., 2008; Garcia et al., 2010; Farmer et al., 2016). In the cerebellum, BG require persistent Shh signaling from PCs to maintain their unique gene expression profile. When Shh signaling is abolished in BG, they take on a VA-like expression profile. Remarkably, activating the Shh pathway in the adjacent VAs drives a BG-like transcriptional profile. Therefore, the major determinant of astrocytic gene expression in the cerebellum is a persistent contextual cue provided by a specific neuron population and not intrinsic programing. In addition to driving the BG transcriptional profile, we found that Shh signaling regulates the expression of Kir4.1 and the electrophysiological properties of hippocampal and cortical astrocytes *in vivo* (Farmer et al., 2016). The ability of differential Shh signaling to drive such profound changes in mature astrocytes demonstrates that astrocytes are far more plastic than previously thought.

### Notch Signaling Represses a Neurogenic Program

Notch mediates a highly conserved cell-cell contact pathway that is essential for patterning events throughout development. In the developing cerebellum, Notch signaling ensures that BG properly elaborate around PCs (Eiraku et al., 2005; Komine et al., 2007; Hiraoka et al., 2013). In the adult brain, astrocytes are enriched in Notch signaling components (Cahoy et al., 2008). Surprisingly, blocking astrocytic Notch signaling in the uninjured cortex causes astrocytes to become neurogenic. Conversely, activating Notch signaling in astrocytes prevents neurogenesis after injury (Magnusson et al., 2014). Therefore, mature astrocytes maintain persistent activation of the Notch pathway to remain quiescent under basal conditions. In addition, the Notch pathway may regulate the expression of several genes including GLT1 (Hasel et al., 2017). The specific ligands that activate Notch signaling in adult cortical astrocytes to maintain quiescence remain unknown.

### Fibroblast Growth Factor (FGF) Signaling Regulates Reactive-Like Phenotypes

The fibroblast growth factor (FGF) signaling pathway is involved in many processes including the morphological elaboration of drosophila astrocytes (Stork et al., 2014) and astrocyte differentiation in rodents (Lin and Goldman, 2009). Adult astrocytes maintain the expression of FGFR1, FGFR2 and FGFR3 (Zhang et al., 2014; Chai et al., 2017). Blocking FGF signaling in adult astrocytes causes astrocytes to upregulate GFAP and display a hypertrophic morphology, the classical indicators of astrocyte reactivity. Driving FGF signaling in an injury model prevents these changes (Kang K. et al., 2014; Kang W. et al., 2014). These findings indicate that astrocytes require persistent FGF signaling to maintain a non-reactive morphological phenotype and basal GFAP levels. Like Notch signaling, the source of the ligand(s) that activate FGF signaling in mature cortical astrocytes have yet to be uncovered. Additionally, it is not known if other genes are regulated by FGF signaling.

## Conclusion and Future Perspectives

Astrocytes are a remarkably heterogeneous population of cells that contain distinct properties unique to the brain regions in which they reside. While developmental patterning events are critical for fostering astrocyte heterogeneity, they cannot fully account for the production and maintenance of such diverse astrocyte types. Consistent with this, studies have now shown that mature astrocytes depend on a number of intercellular signaling pathways to maintain their specific properties by regulating expression of receptor/channel/transporter expression, their quiescence and morphological properties. We currently do not know the full complement of the pathways that drive gene expression in mature astrocytes or how these types of pathways interact to establish specific gene expression profiles. Moreover, there are many questions regarding the origin and specificity of context-dependent signaling underlying astrocyte diversity and under what circumstances this signaling can change. Interestingly, chronic disruption of signaling pathways related to Shh, Notch and FGF in the adult brain leads to astrocyte reactivity which is reminiscent of pathological states in CNS injury/disease. Thus, the ability of astrocytes to detect and utilize environmental cues may serve a dual purpose by: (1) generating further astrocyte diversity; and (2) maintaining healthy, non-reactive astrocyte phenotypes. Further studies are needed to understand the fine balance between these scenarios and reveal potential new mechanisms allowing astrocytes to transition from a healthy to a disease state. Ultimately, understanding how astrocyte heterogeneity is generated will allow us to better understand fundamental properties of the CNS and potentially improve brain/spinal cord function following injury or disease.

## Author Contributions

WTF conceived, researched and wrote this review. KM assisted in writing and editing.

## Conflict of Interest Statement

The authors declare that the research was conducted in the absence of any commercial or financial relationships that could be construed as a potential conflict of interest. The reviewer HH and handling Editor declared their shared affiliation.
